# Context, Consequence, Coincidence, and Cumulative Cultural Evolution: Linking Creativity and Culturo-Behavioral Phenomena Together Using Systems Principles and Processes of Selection by Consequences

**DOI:** 10.1007/s40614-025-00492-y

**Published:** 2026-01-29

**Authors:** Jonathan V. Krispin

**Affiliations:** Department of Management and Marketing, Valdosta State University, Valdosta, GA USA

**Keywords:** Cultural systems, Self-organization, Selection by consequences, Adjacent possible, Culturo-behavioral hypercycles

## Abstract

The present article focuses on presentations from the second topical cluster from the 2024 Theory and Philosophy Conference held by the ABAI—Cultural Systems. The cultural systems cluster was comprised of two primary talks—“Unhinging Design from Darwinian and Skinnerian Selection” (Wasserman, 2024), and a second, co-authored by Sigrid Glenn and Maria Malott (and delivered by Sigrid Glenn), entitled “Behavior and Cumulative Cultural Evolution.” We will begin by briefly summarizing some of the main points of each talk, and then discussing some of the implications of the arguments developed in each. The approach taken to link these two seemingly different primary talks will be interdisciplinary. I will seek to illustrate how dynamic patterns of systemic interactions within systems of physical energy parallel the dynamic patterns of behavioral systems and enable us to “reconstruct” some of the main principles emphasized in the primary talks, while also seeking to develop an understanding of how various processes of selection by consequences (natural selection, operant selection, and selection of cultures) emerge from systemic interactions.

The present article focuses on presentations from the cultural systems cluster from the 2024 Theory and Philosophy Conference held by the Association for Behavior Analysis, International. Cultural Systems was an intriguing name for this cluster for several reasons. First, despite the name of the cluster, the two main invited talks given by (1) Ed Wasserman and (2) Sigrid Glenn and Maria Malott did not explicitly develop the theme of the cluster. Second, although there has been much discussion within behavior analysis of its relationship with systems thinking, this link has not been formalized. In the present article, based on the discussant presentation for the cultural systems cluster, significant consideration will be given to developing the theme of systems in light of the two main presentations.

The cultural systems cluster was comprised of two primary talks—“Unhinging Design from Darwinian and Skinnerian Selection” (Wasserman, 2024), and a second, co-authored by Sigrid Glenn and Maria Malott (and delivered by Sigrid Glenn), entitled “Behavior and Cumulative Cultural Evolution” (Glenn & Malott, 2024). We will begin by briefly summarizing some of the main points of each talk, and then discussing some of the implications of the arguments developed in each. The approach will be interdisciplinary, seeking to illustrate how dynamic patterns of systemic interactions within systems of physical energy parallel the dynamic patterns of behavioral systems and enable us to “reconstruct” some of the main principles emphasized in the primary talks while also developing a comprehensive understanding of how various processes of selection by consequences (natural selection, operant selection, and cultural selection) emerge from systemic interactions.

## The Primary Talks from the Cultural Systems Cluster

Ed Wasserman, in his presentation “Unhinging Design from Darwinian and Skinnerian Selection,” developed an argument initially made by Skinner (see Skinner, 1974) with a goal of debunking the need for purposeful and foresightful design in the expansion of novelty and creativity in behavior, building on the work of Charles Darwin. Darwin used his theory of natural selection to assert that the intricate and complex structures observed in living organisms could develop over time via random variation without the need for referencing an intelligent designer.

In Darwin’s (1887/1958) theory, random variations in the physical attributes of a given organism provide differential survival advantages (and disadvantages) to some organisms over others (whether of the same species or another species entirely) in a common environmental context—survival of the fittest. Those organisms that survive to reproduce pass their physical attributes on to a subsequent generation. The subsequent generation exhibits some similar and some dissimilar characteristics (due, again, to random variation and, we now know, genetic recombination) providing differential survival and/or reproductive advantages over repeated generations. This recurrent process, over extended periods of time, explains the diversity that we see within and between species without invoking any intentional design (or intentional designer).

Wasserman went on to consider Skinner’s similar assertion that other processes of selection by consequences, like operant selection and cultural selection, could perhaps likewise account for the intricate and diverse webs of behavior that we presently observe in the world around us. In *About Behaviorism*, Skinner (1974) said the following:

Evolutionary theory moved the purpose which seemed to be displayed by the human genetic endowment from antecedent design to subsequent selection by contingencies of survival. Operant theory moved the purpose which seemed to be displayed by human action from antecedent intention or plan to subsequent selection by contingencies of reinforcement. . . . In the field of human behavior the possibility arises that contingencies of reinforcement may explain a work of art or the solution to a problem in mathematics or science without appealing to a different kind of creative mind or to a trait of creativity. . .). (pp. 246–247)

Skinner (1974) also argued that:

As accidental traits, arising from mutations, are selected by their contributions to survival, so accidental variations in behavior are selected by their reinforcing consequences . . . the biographies of writers, composers, artists, scientists, mathematicians, and inventors all reveal the importance of happy accidents in the production of original behavior. The concept of selection is again the key. The mutations in genetic and evolutionary theory are random, and the topographies of response selected by reinforcement are, if not random, at least not necessarily related to the contingencies under which they will be selected. (pp. 126–127)

Wasserman’s focus, both in the talk given at the Theory and Philosophy Conference and in his recent book, *As if By Design* (Wasserman, 2021) is on presenting a number of different case studies, such as those to which Skinner alluded above, that demonstrate how strikingly novel and significant changes in behavioral practices could be accounted for by the combination of the current context, coincidental, or chance, occurrences, and finally, selection of the novel behaviors (and/or their outcomes) via the consequences of the innovation—or, as he succinctly describes it: context, consequence, and coincidence.

In defining context, Wasserman referenced the “discovery” of several innovations, notably the Fosbury Flop approach to high-jumping and the development of the theory of natural selection; both innovations were developed by multiple people at the same time. Each of these innovations is largely credited to individuals whose accomplishments achieved notoriety more quickly (Dick Fosbury and Charles Darwin, respectively), but they were not the only ones who developed the innovation at that point in time. To account for these independent and simultaneous innovations, Wasserman notes that the different innovators were operating within similar prevailing contexts. In summarizing what is meant by this, Wasserman references a quote by Cep (2019), who was considering the work of Thomas Edison. Cep wrote: “The problems of the age attract the problem solvers of the age, all of whom work more or less within the same constraints and avail themselves of the same existing theories and technologies.”

When defining coincidence, Wasserman (2021) referred to the effects that chance occurrences have on the creation of behavioral innovations. Coincidental, or chance, occurrences include random variation in a behavior performed by the innovator but may also include the addition of external factors that change the particular (as opposed to the prevailing) context within which the innovator is performing. This might also include serendipitous occurrences placing the innovator in the right place at the right time for the behavioral innovation to occur.

In his talk, Wasserman gave several illustrations from the work of others to support the role that coincidental factors may play in innovation. In one example, Wasserman discussed the development of the sound holes (or f-holes) on violins as outlined by Nia et al. (2015), highlighting how the changes to the shape of these holes were altered gradually over several centuries until they converged on the shape that is commonly seen today. Nia et al. speculated that many of these changes likely emerged by chance, or perhaps due to variation in craftsmanship abilities or other limitations. Those variations that resulted in better quality and/or quantity of sound projection were then selected and repeated.

In Wasserman’s and Skinner’s frameworks of behavioral innovation over time, sources of random variation may have served as initial sources of variation, subjected to selection by consequences (the third “C” in Wasserman’s framework). These random variations were followed by a transition into what Wasserman referred to as “tinkering,” and then into more modern processes of innovation involving engineering. This transition was facilitated by the development of language, enabling first an oral tradition and then written words and detailed diagrams as means of passing knowledge between generations, the foundation of what Skinner (1981) referred to as the third level of selection by consequences, the evolution of cultures.

This leads us to the second talk in the Cultural Systems cluster, “Behavior and Cumulative Cultural Evolution,” co-authored by Sigrid Glenn and Maria Malott, and given by Sigrid Glenn at the conference. Glenn and Malott have each made significant individual and collaborative contributions to the areas of cultural evolution and systems analysis considered from a behavior analytic perspective. In this talk, Glenn and Malott (2024) made a considerable effort to connect the behavior analytic perspective on cultural development and evolution to similar efforts in disciplines external to our field. In particular, they provided a behaviorist perspective on cumulative cultural evolution (CCE), particularly as outlined by Mesoudi and Thornton (2018). Theories of CCE attempt to explain the relative evolutionary success of humans (over other species) by focusing on the processes through which human cultures uniquely accumulate modifications (in behaviors, in tools and technologies, etc.). This is accomplished by building on past practices that may be described as *improvements* and are transmitted or accumulated (and further improved) across many generations.

Mesoudi and Thornton (2018) consolidated similarities among various theories of CCE using four core criteria that must be satisfied for CCE to persist over time. These core criteria are (1) behavioral novelty, or variation over time; (2) transmission of behavior from one individual to another; (3) improvement of the transmitted behavior (innovation) relative to some measure of performance (enhancing inclusive genetic and/or cultural fitness); and (4) repeated innovation and social learning over time, generating sequential improvement in performance.

To the four core criteria, Mesoudi and Thornton (2018) added five additional criteria that they termed *extended criteria*. These were (1) multiple functionally dependent cultural traits; (2) diversification into multiple lineages; (3) recombination across lineages; (4) cultural exaptation; and (5) cultural niche construction. In the first extended criterion, multiple socially learned solutions to improve performance, each of which may have partially improved performance relative to a performance measure, may be chained together across multiple steps, leading to continual improvement on the performance measure over time. In the second extended criterion, one initial lineage can expand into multiple, initially parallel lineages that diverge over time. For example, projectile hunting might originate with rock-throwing, then expand into slings, spears, bow and arrow, crossbow, etc. The third extended criterion involves the combination of individual actions/behaviors into chains of behaviors that produce novel outcomes (“things done”). The fourth extended criterion, cultural exaptation, results when a trait that originally evolved to maximize one performance measure is adapted (exapted) to fulfill another. An example of this might be the use of iron hinges, originally developed to allow cathedral doors to pivot, applied to allow a ship’s rudder to pivot. Finally, the fifth extended criterion, cultural niche construction, results when the process of cumulative cultural evolution modifies and creates its own selection pressure (more on this later).

In CCE, one of the fundamental concepts that is implicitly or explicitly embedded in numerous core and extended criteria outlined by Mesoudi and Thornton (2018) is the concept of lineages. These lineages provide a means for inheritance—passing along a cultural practice through successive generations. Mesoudi and Thornton assert that the first core criterion provides a source of variation, and they make it explicit that the variation required for CCE is *behavioral variation.* The second and fourth core criteria provide an inheritance system—the lineages that are implicit in the first extended criteria and referred to explicitly in the second and third extended criteria. Throughout their talk, Glenn and Malott described several different lineages identified by behavior analysis that might develop as a result of processes of selection by consequences, with each successive lineage constructed by ever-more complex combinations of the basic building blocks of behavior and/or its products. These lineages include operant lineages, culturo-behavioral lineages, culturant lineages, and metaculturant lineages, each of which will be discussed briefly below.

Operants (lineages of recurrence of behaviors of the same class), the most fundamental of the behavioral lineages, may form via asocial (naturally occurring) consequences or socially provided consequences. Operant lineages provide the source of behavioral variation over time from which CCE results (addressing the first core criterion of CCE). When these operants are transmitted from one individual to another, a *culturo-behavioral lineage* may form, consisting of interlocking behavioral contingencies (IBCs), addressing the second core criterion of CCE. As Skinner (1981) has emphasized, in a social or cultural setting, behaviors that may have been initially learned via naturally occurring sources of reinforcement may be transmitted from one person’s repertoire to another’s via socially provided consequences. Regardless of path, practices that proliferate within a given group to sufficient extent and for a sufficient length of time may be labeled *cultural practices.* Glenn (2004) asserted that culture is fundamentally defined by such transmissions of behavior learned by one organism to the repertoires of other organisms. Cultural practices that are maintained by local, operant contingencies often stemming from environmental similarities she termed *macrobehaviors*. Such behaviors can give rise to *macrocontingencies*, creating cumulative consequences that may affect the group overall (e.g., pollution and overconsumption of scarce natural resources resulting from cultural consumption patterns, for example, or the adoption of innovations in projectile hunting practices to which Mesoudi and Thornton (2018) referred in the second extended criterion). When there are cumulative positive effects that collectively benefit the members of the culture, adoption and adaptation of new variations of practices can lead to the kinds of performance improvements that are discussed in the third and fourth core criteria of CCE, for example.

In their talk, Glenn and Malott went on to talk about another type of lineage, the *culturant lineage*. Glenn (2004) argued that these types of lineages emerged via a new level of *cultural selection.* Culturant lineages are formed when IBCs produce an aggregate product (AP) that is selected by a selecting environment re-occur over time. Culturants (named as such as a cultural-level analog of the operant; see Hunter, 2012, or Glenn et al., 2016, for more discussion) are governed by *metacontingencies* stemming from cultural consequences that apply across all of the IBCs involved in the production of the AP, leading to their recurrence. This kind of chaining of behaviors via IBCs aligns closely with several of the extended criteria that Mesoudi and Thornton (2018) identified. Their first extended criterion specifically referred to chains of *partial solutions across multiple steps* that lead to continual improvement of performance relative to a performance measure, for example, whereas their third extended criterion focused on recombination across lineages into chains of behaviors that produce novel outcomes. Both of these patterns in cultural evolution may be realized in culturant lineages, and via the last type of lineage that Glenn and Malott describe—the metaculturant lineage.

The progression of lineages that Glenn and Malott described culminated with lineages of metaculturants; culturants can combine to form recurring metaculturants producing multiple aggregate products (this might include the relationships and exchanges between supplier and producer companies in a business process/supply chain, for example). This type of lineage forms when the AP of one culturant is selected by another culturant, serving as an input into the IBCs of the second (or third, or fourth) culturant included in the metaculturant. The cumulative cultural consequences produced by these culturant linkages result in their recurrence and the development of a metaculturant lineage that persists over time.

The last concept that Glenn and Malott discussed in their talk was not another lineage but rather was a concept developed to address factors that might interact to facilitate the emergence of significant cultural change, a phenomenon known as the *cultural cusp*. The cultural cusp was initially defined by Glenn et al. (2016) in the following manner: “Nonrecurring interlocking and/or individual behavioral contingencies that coalesce to produce an aggregate product that gives rise to significant sociocultural change.” Although this definition has been used (and useful) in subsequent discussions and investigations into large-scale cultural changes, Glenn and Malott (2024) put forth a revised definition for the cultural cusp as follows: dynamic ecosystems resulting in a novel product that leads to significant cultural change in social, political, and economic environments. They argued that each of the ever-more complex lineages that they outlined in their talk (and which we have discussed above) form the various elements that might interact in these dynamic ecosystems. We will return to this discussion later in this article when we explore a third process of selection by consequences—the *selection of cultures*.

## Selection by Consequences

Although these two talks were distinct in the topics that they covered, both also have some areas of convergence, especially in the importance of selection by consequences. In his article, *Selection by Consequences*, Skinner (1981) argued that “selection by consequences is a causal mode found only in living things, or in machines made by living things” (p. 501). He asserted that selection by consequences presumably started with the emergence of a molecule that could reproduce itself, with reproduction itself being the first consequence that led to the first level of selection by consequences, natural selection. Natural selection (the first level of selection by consequence) presumably then led to the evolution of cells, organs, and organisms, all of which possessed the characteristic of being able to reproduce themselves, thus subjecting them to different forms of selection by consequences.

A second kind of selection by consequences is operant conditioning, emerging due to a “susceptibility to reinforcement by certain kinds of consequences and a supply of behavior less specifically committed to eliciting or releasing stimuli” (Skinner, 1981, p. 501). Although natural selection might select some behavioral traits (those behaviors that are passed from generation to generation via genetic inheritance and provide some survival advantage to a species will continue due to the survival of the species), Skinner argued that operant selection may both supplement natural selection of behavior and may even supplant it as the primary means through which behavior is selected. This is one of the fundamental premises of the field of behavior analysis—behavior is a function of its consequences (Glenn, 2004).

Skinner (1981) subsequently went on to argue for a third level of selection by consequences, responsible for the evolution of social environments or cultures. In Skinner’s description of this level of selection, he asserted that it began at the level of the individual when a learned behavior (learned via operant conditioning) resulted in a new practice that provided some survival advantage to the individual. An example of such a practice might include learning a better way to grow food, resulting in a larger production capacity. These practices may spread throughout a social group by means of both socially provided reinforcement, and/or naturally occurring reinforcement (see Glenn, 2004). However, Skinner emphasized that the culture, which he elsewhere defined as a set of contingencies of reinforcement maintained by a group (Skinner, 1974), evolves when its practices successfully solve the problems that it is facing, thereby benefitting the group overall.

### Criteria for Processes of Selection by Consequences

Now that we have at least initially defined the three fundamental processes of selection by consequences as articulated by Skinner, we will give some consideration to developing an understanding of the key factors that make up such processes. Such processes are the heart of differential selection of one species over another, one behavior over another, one cultural practice over another, and ostensibly one culture over another.

Zeiler (1992) asserted that processes of selection act on outcomes but select the processes (or mechanisms) that produce them. Given this, we must first have a process through which the outcomes that are candidates for selection are produced. Second, differential selection is predicated on the occurrence of *variability* in the outcome of the production process (see Sandaker, 2009) due to variability in the inputs to the production process, and/or variability in the particular activities in the production process. Third, there must be some potential for the recurrence of the production process (and reproduction of its outcome, as Skinner (1981) emphasized), and finally (fourth) there must be some form of reciprocal influence between the production process and its selecting environment (Marr, 1996; Mattaini, 2007). The potential for recurrence and reciprocal influence are necessary conditions for a *lineage* of interactions between the producing system and its environment over time, leading to the potential for evolution and adaptation (see, for example, the discussion of lineages presented in Glenn, 2004).

### Processes of Selection as Dynamic Systems

Of the factors included in a process of selection by consequences, the fourth factor merits particular attention—the reciprocal influence between the selecting environment and the production process. This reciprocal influence may be based on material exchanges between the selecting environment and the production process (like changes in access to resources needed by the production system due to consumption over time) but may also come via other forms of contact between the production system and changes to its environment produced by the production system. For example, such exchanges may form the basis of the consequences that govern behaviors at the operant level. In either case, the reciprocal influence forms the basis for *feedback* between the producing process and its selecting environment, making the production process, should it repeat, more than a recurrent process; feedback makes it an *iterative* process. An iterative process uses outputs from the previous cycling of the process as inputs into subsequent cyclings—it picks up where it left off, making iterative processes more than “just” processes—they are *systems.* Selection by consequences in any of its forms is thus a systemic phenomenon.

Feedback is one of the fundamental characteristics of systems. One of the fundamental differences between a *process* and a *system* is the presence of feedback—feedback is an added element that elevates a process to the status of *system*. It is the feedback structure that determines the particular change dynamics (or “behavior” in a general sense) of a system, and all learning depends on feedback (Sterman, 2000). There are two fundamental types of feedback: positive feedback (sometimes referred to as amplifying or self-reinforcing feedback) and negative feedback (sometimes referred to as balancing, or goal-seeking feedback). Positive feedback accelerates the rate of change within a system leading to exponential growth (or exponential collapse) and often leads to instability and/or catastrophic destruction in physical systems if/when the rate of activity accelerates to an unsustainable rate. Negative feedback decelerates the rate of change within the system, and, in many cases, stabilizes a system within which there are also positive feedback dynamics present.

## Combinatorial Systems and the Rise of the Relevance of Context

Now that we have a basic understanding of processes of selection by consequences and their link to systems and system dynamics, we will return to the framework of context, coincidence, and coincidence that Wasserman (2021, 2024) asserted was responsible for the diversity that we observe in the behavioral realm. We will examine this framework through the lens of complex systems, and particularly the theory of self-organizing systems. Defining what is meant by “complex systems” is notoriously difficult and there has not yet been a definition that has achieved consensus agreement (Bianconi et al, 2023). *Journal of Physics: Complexity* published a special issue in 2023 celebrating the awarding of the 2021 Nobel Prize in Physics to several researchers for contributions to theories related to complexity in physical systems. As part of this issue, the members of the journal’s editorial board were asked to provide their own definitions of complex systems. One member refused to answer the question, instead choosing to provide criteria that might be used to identify complex systems (Bianconi et al., 2023). These systems always exhibit several characteristics including (1) having many interacting constituents; (2) exhibiting the property of collectivity; (3) exhibiting nonlinearity in the interactions between constituents; (4) having feedback mechanisms that affect the dynamics of the system; and (5) exhibiting emergent properties. Further, such systems frequently display some or all of the following properties: (1) structures that exhibit a hierarchical organization; (2) displaying an ordering of relationships among constituents; and (3) exhibiting the property of adaptivity.

The characteristics of many interacting constituents and demonstrating nonlinear dynamics are properties that are commonly exhibited in systems constructed with combinatorial objects. Theoretical physicist Sara Imari Walker (2024) defined combinatorial objects in an interesting context—the search for the evidence of life on distant planets. Her definition of combinatorial objects is as follows, “all things that can be built from elementary building blocks using operations consistent with the physics of our universe” (p. 91). These combinatorial objects begin at the level of chemistry but also include biological objects (like life!) and objects constructed by living systems (like screwdrivers—her actual example). If we accept Walker’s definition of combinatorial objects, behaviors and the products they produce, such as those discussed in the lineages described by Glenn and Malott (2024), are combinatorial objects.

Following her definition of combinatorial objects, Walker (2024) went on to describe several properties that combinatorial objects possess. They are finite and distinguishable, and they are countable as a result. Combinatorial objects are rare, particularly when we compare the combinatorial objects that have actually been made with the enormously larger set of all possible objects that might be constructed. In fact, she argues that the set of all possible combinatorial objects can never be actually constructed because we don’t (1) have enough time, or (2) enough “stuff” (physical matter) to make every possible combinatorial object. Another property of combinatorial objects that might seem obvious is that they can be combined and “deconstructed,” and Walker asserts that examining the subcomponents of more complex objects will give us a glimpse into their history. We may even be able to infer the path to the construction of complex objects since their constituent parts provide a “physically instantiated memory” (p. 90).

### The Adjacent Possible and Coincidence in Combinatorial Systems

In combinatorial systems, the trajectory of the system through its possible states as its constituents interact and combine involves moving into what Kauffman (2000) called the *adjacent possible*. For any given state of a system, its adjacent possible is the set of all possible interactions and combinations, given the current constituents. Expansion of a system into its adjacent possible is itself a selective process (albeit one that might not always rise to the level of selection *by consequences*). As some of the possible combinations become actual combinations, the system “chooses” from among its possible states and “selects” the next actual state in its evolution over time (we will explore more on the processes that produce these “choices” and “selections” in subsequent sections). As this happens, some basic constituents may become absorbed into other, combined units, while those initial basic constituents themselves become “extinct”; some future states that were once possible may become no longer possible, whereas other future states that were once not possible become possible. The system expands into an ever-more constrained set of what was once possible, while simultaneously creating an ever-expanding set of new possibilities based on the potential combinations of the newly formed constituents and remaining/previously existing constituents until some state of maximal diversity is achieved.

To illustrate how this works, we can use factorial expansion of possibilities in a system of a limited total number of combinatorial objects. For example, let’s imagine that we have an environment with 100 discrete combinatorial objects, but among those objects, we find that these objects are initially of only ten different types. With 10 different types of objects, those objects may be combined 3,628,800 different ways (10! = 3,628,800—the actual possible diversity is far greater than this if we allow for the inclusion of multiple instances of objects of the same type in the new objects that may be formed). If we move one step into the adjacent possible, we will “choose” only one path from those possible paths forward. If this choice results in a new, more complex item, then our new current state will have 11 different objects, but the system now has fewer total objects because some of the original 100 objects have now been included in the making of a new, more-complex object. The new total possible combinations in the adjacent possible of this evolved system has increased dramatically however, given that there are now 39,916,800 possible combinations (11! = 39,916,800) of the 11 different types of objects found within the system. However, as the system advances into its adjacent possible, it may actually reach a theoretical limit where the diversity in the system is only one type of object if all the actual objects in the system are combined into one complex object. With only 100 discrete objects in our set, we can never actually simultaneously build all possible combinations.

Walker and her colleagues are building a theory of astrobiology called *assembly theory* that uses combinatorial mathematics to argue that complex combinatorial objects at the molecular level—those that take more than a minimum of 15 steps to construct from chemical elements (Marshall et al., 2021)—are fundamentally rare and can only be constructed (assembled) by constraints imposed by processes of selection, especially when the same complex object (or, more generally, many similar objects of the same class) appears in large numbers. Without a process of selection, the probability of recurrence for the same complex object would be vanishingly small. Imagine a system like the system that we have just described in the paragraphs above with 10 different types of combinatorial objects, but a larger number of total objects in the system—perhaps 1000. What if we observed this system as it moved into its adjacent possible and found that it progressed in such a way to form 100 copies of the same complex object? This would cause us to make an inference—something strange is happening given that the combinations that actually emerge aren’t in conformance with what we would have predicted if only random combinations of the objects in the system had formed. It is implausible for such a phenomenon to occur due to random fluctuations, so some process of selection must be present for this to occur. Walker and colleagues are using this rationale to justify the use of mass spectrometry to analyze chemical compositions in the search for evidence of life on distant planets (looking for large numbers of very complex objects), but this is essentially the same reasoning for which Wasserman (2021, 2024) advocated in behavioral evolution based on interactions among context, coincidence, and consequence. In order for the balance between variation and recurrence via lineages that we observe around us to be possible, all three of Wasserman’s factors must be present.

### Moving from Complex Combinatorial Objects to Complex Combinatorial Systems

As we continue our consideration of complex systems, we will repeatedly refer to two complementary perspectives through which we will consider these systems. These complementary perspectives seek to identify and define the interrelationships between what we will refer to as the *mechanics* of the system, and the *dynamics* of the system. Perhaps the best way to initially describe the distinction between these two perspectives is to examine how they are differentiated in the field of classical (or Newtonian) mechanics, the particular subfield of physics that nicely describes and accounts for the interactions between macroscopic elements moving at relatively slow speeds (at least in relation to the speed of light). Classical mechanics is typically divided into two complementary parts, known as kinematics (or what we are more generally calling *mechanics*) and dynamics. As we will see, the context as Wasserman defines it is related to the mechanical description of a system, whereas consequences as Wasserman defines them, are related to the dynamic description of a system.

The kinematic description of such systems describes the particular elements that are interacting within the specific system under consideration in terms of the characteristics of the constituents and dimensions that relate to their interactions and movements. These are the details that define the particular context of the system, and include such characteristics and properties as mass, displacement (which occurs when an object is moved through space), time, velocity, and acceleration of each particle in the system. For example, the classical ideal gas model would begin with the kinematic description of the objects in three-dimensional state space of the system in terms of their position in each of the three spatial dimensions, and their vectors of motion along each of the spatial dimensions. In ideal gas models, the elements are all assumed to be of identical mass and shape (known as point-particles), but in the real world, the kinematic description of classical systems would have to include other properties like the mass of each element in the system.

In classical mechanics, the complement to kinematics is dynamics. Dynamics is concerned with the description of the causes that account for the changes in the kinematic description over time. Hence, in the realm of classical mechanics, dynamics focuses on forces, momentum, energy, and work, for example. The goal is to uncover lawful relationships that describe and predict the movements (i.e., the dynamic changes) within the system. Newton’s laws of motion would fall into this realm, as would the first two laws of thermodynamics (the laws of energy conservation and entropy production). Although a given kinematic description only holds for one particular system, the principles and lawful relations that are derived from the dynamic description generally govern all kinematic systems to which they apply. For example, Newton’s laws of motion apply to all physical systems for which the constituents are macroscopic and moving at slow speeds relative to the speed of light, as stated above.

When we move into the realm of combinatorial systems of physical energy, the classical kinematic and dynamic distinctions still apply, but, if we want to fully describe the changes in the system over time, we need to expand the factors that we include in our mechanical description to account for additional relevant factors. In chemical systems, for example, this would include properties such as the number and type of the chemical bonds within a molecule, its electrical charge, the particular shape of the molecule in three-dimensions, etc. because all these properties must be considered when seeking to understand which molecules might combine with other molecules, forming a new product.

Likewise, we have to expand the factors considered in our dynamic description of chemical systems. As we understand the causes and forces underlying the combinations possible given our mechanical analysis, we will identify new laws/rules and principles that begin to describe the forces that govern the interrelationships between elements in the system. For example, in chemical systems, we can expand our understanding of dynamic terms such as equilibrium (and nonequilibrium), we can define equations that allow us to determine the rates of reactions from concentrations of reactants, and we can expand our dynamic description of the components involved to include various roles that appear across multiple various reactions, like reactants and products. We can also highlight dynamic patterns such as positive and negative feedback that result from various interactions produced by molecules that influence the dynamics of reactions (like inhibitors and catalysts).

Finally, we can apply the distinction between mechanics and dynamics (as we are defining it here) in the realm of behavioral systems. For example, Catania (1973) differentiated between descriptive and functional operants. In this differentiation, descriptive operants are typified by the definition of the response class as often found in the methodological sections of experimental reports. We describe the behavior in terms of its physical properties—a lever press or a disc peck of some given force, performed at a given rate for example. This type of description would constitute a mechanical description of the behavior in our current conceptualization. Catania went on to define the functional usage of the term operant as those described in the theoretical sections of an experimental report. In that usage, the *response class* is defined as a sequence of behaviors over time that is modifiable by response contingencies such as occur when *reinforcing* or *punishing* consequences are arranged in the presence of *discriminative stimuli* for behaviors of the descriptive class. This second usage occurs within the context of a *functional analysis* (see Zeiler, 1992) and places the responses class in a functional arrangement in relation to other events in its environment. This functional usage of operant moves it into the realm of a dynamic description using terms that can be applied across multiple contexts involving behavioral interactions and has led to defining new dynamic laws that govern behavior (like the *matching law* for example).

For chemical systems, Kauffman (2000) described the various factors that determine the potential and likelihood of a reaction (such as the molecule mass, velocity, electrical charge, physical shape, etc. of the molecules involved) as the dimensions, or degrees of freedom, in the “task space” involved in the combination of chemical elements. The specific properties that constitute the criteria that determine whether a given reaction may occur can be described as the *fitness criteria* for the reaction. Fitness defined in this manner is one of the unique properties of combinatorial systems. Because the particular characteristics of each constituent that determine their relative fitness for inclusion in combinatorial interactions within a system are defined as part of the mechanical description of the system, they are unique/particular to each individual system. Here, we begin to see the relevance of what Wasserman (2021, 2024) referred to as context. A particular constituent may have particular characteristics that enable it to combine in one system, but those characteristics may not be relevant in other systems. In other words, the fitness criteria are contextually defined, and such context-dependent factors become relevant only as we move into the combinatorial realm.

Kauffman (2000) noted that, in biological systems, the relevant *function* of an organ, can only be identified in the context of the larger system of which it is part. The heart, for example, observed in isolation, exhibits a number of different “behaviors.” It emits a regular beating sound. It contracts and expands in a rhythmic manner. The relevant function of the heart, however, can only be determined in the context of the “whole” in which is participates—the primary function of the heart is to pump blood to and from the other organs and cells of the organism in which it exists, providing oxygen and nutrients, removing waste, etc. The relevant dimensions of this primary function determine the dimensions that must align with the selection, or fitness, criteria (or what Mesoudi & Thornton, 2018, referred to as the *performance measure* in their framework of CCE) in order for selection by the receiving system to occur.

Zeiler (1992) observed that the relationship between the primary function of a process (its *immediate* function in Zeiler’s terms) and its general relatedness to the long-term survival of the larger “whole” of which it is part (its *evolutionary* function in Zeiler’s terms) is an “open question” (p. 419), not a foregone conclusion. This distinction aligns closely with the distinction that Skinner (1988) made in the analogy that he drew between the evolution of an *organ* (like the heart that we have been discussing) and the evolution (and survival) of the *organism* (the larger whole in this context) of which it is part and the evolution of a cultural practice and the evolution (and survival) of the culture of which it is part. The immediate contribution made by the organ to the organism (or cultural practice to the culture, or group) is its immediate function, and “improvements” are achieved when the function is improved in its fitness in relation to its immediate function. The long-term contribution that this immediate function makes to the future survival of the organism (or culture/group) due to its *fitness enhancement* with the larger environmental context within which the whole (be it an organism or a culture) is operating is its evolutionary effect. However, there are times when a local improvement does not result in a survival advantage, and there may even be times when a local “improvement” in an immediate function has detrimental effects on the survival of the whole. On a cultural level, the ability to harness the energy found in fossil fuels, for example, has led to enormous economic and quality of life benefits, but has also led to macrocontingent effects like pollution and resource depletion that threaten long-term survival.

## Self-Organizing Systems

The present discussion of the relationship between fitness and function further addresses the emphasis in CCE criteria on fitness as mentioned in both core criteria (numbers 3 and 4) and extended criteria (number 1 and number 4), but we haven’t yet talked about the fifth extended criteria, cultural niche construction. As we move forward, we will develop a behavior analytic theory of evolution at the cultural level that will link cultural evolution and cultural niche construction together via a higher-level process of selection by consequences—a process for the selection of cultures. The development of a new level of selection by consequences governing the *selection of cultures* will be built upon principles of *self-organizing* systems of combinatorial objects developed by Nobel-Prize winning thermodynamicist Ilya Prigogine and his colleagues.

Self-organization is a phenomenon that has been defined as the emergence of structures of a higher-level of organization with a minimum of specific external interference or direction (Hudson, 2000). These emergent structures include the development of functional, spatial, and/or temporal patterns that coordinate across the constituents, or members, of a system, producing much longer-range correlations across these constituents than were previously observed. The occurrence of self-organization is determined based on the dynamics of the interactions between constituents within a system and can occur in systems that have enormous differences in their mechanical constitution. Self-organizing systems define their own boundaries according to their internal dynamics. Like other complex systems, self-organizing systems exhibit nonlinear relationships between changes in their boundary conditions (known as perturbations) and corresponding changes in their internal dynamics and interactions, with the systemic response resulting much more from the internal dynamics of the system than the particular dimensions of the external perturbation. For example, a large perturbation may produce only a small and momentary change in the internal dynamics of the system in some cases, and in other cases, a small external perturbation may result in large-scale, and lasting systemic changes within the self-organizing system. As a result of these nonlinear relations between the system and its environment, self-organizing systems have been described as resistant to external control (see Hudson, 2000, or Mattaini, 2006, for more discussion), but these nonlinear patterns have also been identified as a source of adaptation in self-organizing systems. For example, in a report to the European Commission entitled *Self-Organization in the Physico-Chemical and Life Sciences*, Biebracher et al. (1995) asserted, “Self-organizing systems allow adaptation to the prevailing environment, i.e.,, they react to changes in the environment with a thermodynamic response which makes the systems extraordinarily flexible and robust against perturbations from outside conditions” (p. 1).

Ilya Prigogine, a Belgian thermodynamicist, was awarded the Nobel Prize in Chemistry in 1977 for his work on self-organization in dissipative systems. Dissipative systems are nonequilibrium systems in which energy is flowing, and the specific structures that they exhibit are known as dissipative structures. In some dissipative systems, given the presence of certain necessary conditions, it is possible for organizations of greater order to emerge spontaneously and be sustained over time. There are four necessary conditions: (1) The system must be an open system, exchanging energy, material, and/or information with its external environment; (2) It must be sufficiently displaced from equilibrium; (3) It must exhibit the nonlinear relationship between forces (perturbations at its boundaries, and fluctuations, or deviations from equilibrium, within its boundaries) and fluxes that we mentioned above; and (4) It must be able to accelerate/amplify those perturbations and/or fluctuations (see Prigogine, 1980, 1996; Prigogine & Stengers, 1984, for more discussion). These conditions, and their presence within combinatorial systems (like chemical systems, and behavioral systems), play a crucial role in self-organizing phenomena, and may help us to identify a new process of selection by consequences that accounts for the evolution of cultures, so it is to this topic that we will next turn our attention.

### Self-Organization in Chemical Systems

Because we have already given consideration to chemical systems as examples of combinatorial systems, we will begin with a brief overview of self-organization as it occurs in chemical systems before transitioning to a discussion of how self-organization follows similar dynamics in behavioral systems. As a general result, the rates of chemical reactions are dynamically described by nonlinear equations, but the vast majority of chemical equations in closed systems proceed at an ever-diminishing rate, slowing as the reactants interact and are transformed into the products of the reaction and the system approaches equilibrium. Such dynamics are demonstrative of negative feedback in such systems, whereas the fourth condition of self-organization that we outlined above *requires positive feedback dynamics* that accelerate the rate of activity within a system and amplify fluctuations within a system (which are typically defined as displacements from equilibrium or steady state dynamics) leading to the prerequisite instability in the system organization that must precede potential self-organization. However, there is a class of chemical reactions where it is possible for the rate of the reaction to accelerate—catalyzed reactions. Prigogine and Stengers (1984) asserted that *catalytic loops* are required for such instabilities to occur in chemical systems.

With this information at hand, we can explore several ways in which catalysis can result in catalytic loops as identified by Stuart Kauffman (2000). He didn’t not use the term “catalytic loops” that Prigogine and Stengers (1984) used, instead referring to *catalytic closure* in sets of reactions. The first, and simplest of these is the autocatalytic reaction, defined as a reaction in which a product of the reaction also serves to catalyze the reaction. The second case of catalytic closure occurs when a product of reaction A catalyzes a second reaction (reaction B), and a product of reaction B, in turn, catalyzes reaction A. Finally, in the general case, catalytic closure may be achieved by a set of chemical reactions when the following criteria are met: each reaction in the set is both catalyzed by the product of another reaction in the set *and* also produces a product that catalyzes another reaction in the set, forming a set of reactions that achieves catalytic closure that Kauffman called collectively autocatalytic sets.

### Characteristics of Closed Autocatalytic Sets

Such sets, however they are particularly structured, meet all the criteria and characteristics that we have previously described relating to self-organized systems. They define their own boundaries, for example, based on the definition of inclusion for each constituent in the set. If a given reaction is both catalyzed by a product of another reaction in the set *and* itself produces a product that catalyzes another reaction in the set, then it is a member of the set, and the set of all such reactions forms the system. Those reactions occurring in the same environment that don’t meet these criteria are not part of the system, although they may create effects that influence the dynamic behavior of the system. The definition of the set also that we outlined above demonstrates a characteristic that Ulanowicz (2009) has called *irreducible wholeness*. If even one reaction is left out of consideration, the wholeness of the set is not established. Each member of the set both produces a product that catalyzes another reaction in the set *and* is itself catalyzed by the product of another reaction in the set. If one reaction is excluded, then the criteria that determine the boundaries of the set cannot be identified.

Kauffman (2000) has argued that the emergence of these sets becomes inevitable as a given chemical system reaches sufficient diversity based on the fact that just about any molecule has some probability of serving as a catalyst for other reactions. For example, in the realm of proteins, research has shown that the probability that a given peptide (a small protein made of short chains of six amino acids) can serve as a catalyst for the ligation of a protein is in the realm of one in roughly 1 trillion or so. Once the diversity of proteins reaches 1 trillion different proteins, then we cross a threshold where it is likely that there is another protein in the system that will catalyze every possible reaction. Smaller collectively autocatalytic sets of proteins will emerge before that level of diversity. For context, it is common for chemists to produce a diversity of 100 trillion proteins in a single test tube.

The structures that are formed in collectively autocatalytic sets thus appear with “apparent spontaneity” as the system crosses these transition thresholds and, once a collectively autocatalytic set of reactions emerges, it exhibits structures of greater order. The activity of the set of reactions within the system become highly correlated and coordinated, and this coordinated activity is able to persist over much longer time spans than is common for a typical reaction. Such sets can, for example, achieve sustained and stable rates of activity (a *dynamic equilibrium*) if the system is open to its environment and has access to sources of food/fuel, which, in this case, consists of sufficient amounts of the reactants to sustain the reactions in the set. The system also exhibits nonlinear responses to perturbations at its boundaries and the dynamic response of the system to such perturbations are determined by its internal structure rather than by the direction and magnitude of the perturbation. So, such sets are self-organizing.

Not only are these sets self-organizing, but they also facilitate their own reproduction. Each reaction in the set is catalyzed by the product of another reaction in the set, thus *differentially selecting all the reactions in the set in comparison with other reactions in the environment* (not included in the set). This collective differential selection is evidence that there is a *new level of selection by consequences* defined by the dynamics of the set. This phenomenon is evidenced by another characteristic of such sets, *centripetality* (Ulanowicz, 2009), and stems from the fact that the collective rate of the reactions in the set will increase until it reaches some physical constraint. For example, the collective rate of reactions will accelerate until there is a catalyst for every instance of the reactions involved in the set, at which point the collective rate of reactions will plateau. This collective, continual acceleration of the reactions in the set demonstrates the positive feedback dynamics present within the set necessary to meet the condition for self-organization outlined by Prigogine and his colleagues. The other physical constraint that may constrain the collective rate of reactions is the availability of “food” to sustain the reactions in the set. If even one reaction is limited by the availability of the reactants in the reaction, then the collective rate of reactions will be governed by that limitation.

The fact that the set of reactions is continually elevated and sustained over time by merit of its structure makes it potentially amenable to various other processes of selection. We mentioned above that the collective differential selection of reactions (evidenced by the sustained and elevated collective rate of the reactions) defines one new process of selection by consequences. Another process of selection by consequences occurs *at each niche created within the set*. In this case, a niche is defined as the particular criteria that define the fitness of each reaction product in serving as a catalyst for another reaction in the set. A particular catalyst may be replaced by a product of another reaction that “fits” the catalyst task space better than the original catalyst. In such cases, a reaction initially included in the set may be displaced by another reaction. In other cases, where the collective rate of reactions is governed by a limitation caused by a lack of availability of reactants in one reaction, that product/catalyst may be supplemented by a product of another reaction that may also catalyze the constrained reaction, leading to redundancies within the set. In such cases, the collective, centripetal acceleration of the set increases the top-down, selection pressure that the set imposes on its surrounding environment, forming the basis for “competition” among other reactions (and their products) in the environment for inclusion in the set.

### The General Occurrence of Collectively Autocatalytic Sets in Systems of Combinatorial Objects

The basic principles that govern collectively autocatalytic sets have been extended into other types of combinatorial systems by Kauffman and others. For example, Kauffman (2000) himself extended these principles into the economic realm, translating autocatalysis into what he termed *advantages of trade*. Ulanowicz (2009) extrapolated these principles into the ecological realm, generalizing autocatalysis to autofacilitation and mutualisms. Krispin (2017, 2019) extended these principles in the behavioral and culture-behavioral realms by connecting Ulanowicz’ concept of autofacilitation with reinforcement, arguing for the emergence/self-organization of culturant and culturo-behavioral, hypercycles.

On the level of the metacontingency, when we define the particular interlocking behaviors (IBs) included in the chain of behaviors, and when we describe particular characteristics of the AP produced by those IBs and the contribution that it makes to the receiving system that selects it (its immediate function in Zeiler’s [1992]) terms, we are describing the mechanics of the process and the mechanical characteristics of the AP that results in the differential selection of one AP over another. These characteristics result in the definition of the fitness criteria for selection by the receiving system which we might refer to as *selection contingencies* and therefore falls into the mechanical description of the process or system. However, when we are discussing the contingencies (IBCs) that govern the recurrence of the IBs in the context of the culturant, and the terms that describe the influence of these dynamic influences (like *reinforce* or *facilitate*) during their interactions, we are working in the dynamic realm of the system. This dynamic description would include *operant contingencies* when we are examining individual behaviors and *metacontingencies* (see Glenn, 2004, and Glenn et al., 2016) when we are examining culturants.

The “receiving system” may be the individual that performed the behavior (behavioral consequence) but may also be an external receiving system (as is the case in many metacontingent relationships). The criteria used by an external receiving system when selecting an aggregate product are connected to the mechanics of the producing process and its adaptation over time—explaining in behavioral terms what Mesoudi and Thornton (2018) referred to as the *performance measure.* The outcome can be modified to improve in relation to these selection criteria. In behavior analysis, defining these criteria and modifying the behavior (and its outcome(s) to address these criteria) involve processes like shaping and chaining behaviors in applied behavior analysis, and pinpointing (Daniels, 1989) in organizational behavior analysis. The behavioral consequences (like positive reinforcement, negative reinforcement, and punishment) experienced by the producing system upon selection determine the dynamics of the system via the feedback dynamics involved. This combination of the mechanics and dynamics of a behavioral system form a complementary dual that will play a critical role in subsequent discussion, and in the development of a higher-level process of selection by consequences.

Of course, the functional analysis of behavior expressed in the operant contingency is very different than the description of the immediate and evolutionary functions of those same behaviors. These considerations are essentially independent of one another. The selection outcomes that reinforce a given behavior may be related to its immediate function and fitness with the organism’s environment, but also may be unrelated, or even dysfunctionally related to those functions (counterproductive). This can result in a decoupling of the consequences that sustain a behavior from the impacts that those same behaviors can have on both the immediate and long-term survival of the organism that exhibits the behavior—the “open question” between immediate and evolutionary functions that Zeiler (1992) posed. This disconnect can likewise occur at the cultural level when cultural practices proliferate despite reducing the likelihood of survival of the cultures that adopt them, due to cumulative, adverse *macrocontingent effects*. The selection (via reinforcement) of a behavior in the repertoire of an individual is often the result of different processes of selection than those that select the outcomes of the behavior in relation to the external environment (evolutionary selection based on survival, for example).

## Discussion

The next several points may be obvious at this juncture, but we need to make the implicit explicit as we move forward and hopefully converge on some logical conclusions. First, behaviors and the outputs of behaviors are combinatorial objects. Behaviors can combine mechanically to produce the outcomes/products that are selected by other receiving systems (or even by the individual that produced the output) due to their fitness in performing a function (like Zeiler’s [1992] immediate function) in relation to its selecting environment. Behaviors can also combine dynamically, as is the case of the three-term operant contingency and the functional analysis of antecedent–behavior–consequence demonstrates. The reinforcing consequences experienced by the producer(s) of the behavioral outcome upon its selection by a receiving system (mechanics) leads to the recurrence of that/those behaviors—a causal/dynamic account of the occurrence/recurrence of the behavior over time.

Second, behaviors and their products expand into their adjacent possible. The behaviors and the products that currently exist in the prevailing and particular contexts (and only those) can combine to form new, more complex behavioral constituents via processes of *melioration*, not optimization. The concept of melioration has long been understood in behavior analysis—as the matching law demonstrates, behavior is allocated among locally available sources of reinforcement, with “a moment-to-moment tendency to shift toward higher local rates of reinforcement. . .” (Herrnstein & Vaughn, 1980, p. 164).

As Glenn and Malott (2024) demonstrated via their analysis of CCE and Wasserman (2021) argued in his analysis of context, the specific content present within the system in the form of behavioral repertoires and aggregate products determines what combinations are possible, and it is the consequences experienced by both the producing and selecting organisms (and/or organizations) that select from among the combinations that are tried which ones will continue on into the adjacent possible. The progression from operant lineages to interlocking behavioral contingencies to socio-cultural lineages, to culturant lineages and finally to metaculturant lineages are a necessary result of the processes through which combinatorial behavioral systems expand into their adjacent possible states over time.

**Fig. 1:**
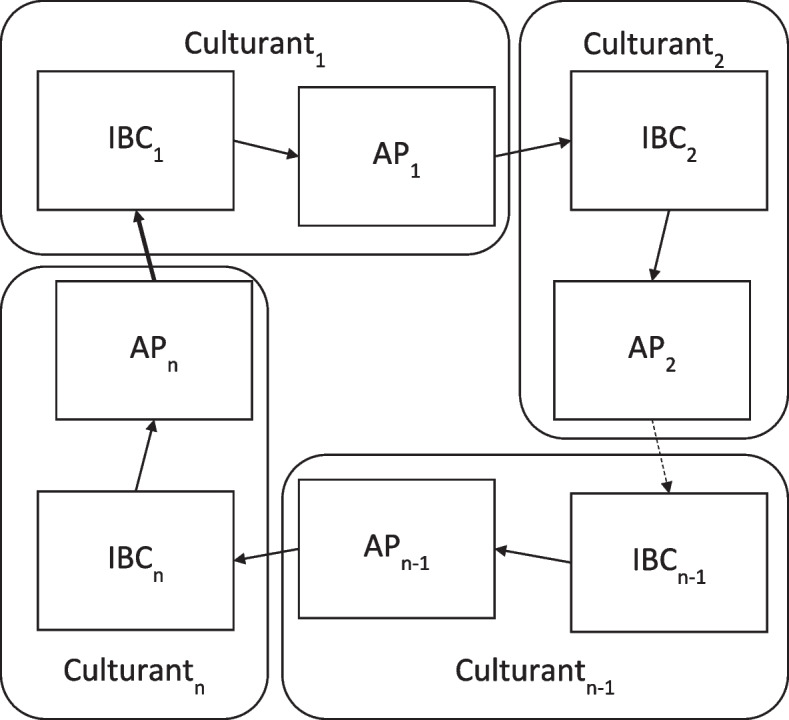
The Culturant Hypercycle. *Note*: In general form, IBC_1_ produces AP_1_, selected by IBC_2_ that produces AP_2_ . . . selected by IBC_n-1_ that produces AP_n-1_, selected by IBC_n_ that produces AP_n_ that is selected by IBC_1_. . . . As a result of the collective dynamics of the set, all of the culturants in the set are differentially selected as a set (group), promoting this group of practices over other practices that are outside of the set (between-group selection; Krispin, 2019)

In parallel to Kauffman’s emergent sets of collectively autocatalytic reactions, we can likewise have the emergence of sets of collectively-facilitative culturants—*culturant hypercycles* that are even one step further into the adjacent possible than the metaculturants proposed by Glenn and Malott (2024). As Krispin (2019) explained, such sets emerge when we have a set of culturants such that each culturant in the set both produces an AP selected by another culturant in the set (due to its mechanical fitness) *and* itself selects an aggregate product from another culturant in the set as an input into its process (see Figure 1). When the selection of the AP provides an advantage to the selecting culturant for each member of the set (in relation to other similar culturants in the environment), a positive feedback loop is established and the property of centripetality is demonstrated—the rate of occurrence of all members of the set is collectively accelerated. In other words, all culturants in the set are differentially selected (in relation to other culturants and metaculturants that may be present in the environment)—a new level of selection by consequences. Further, at each exchange between constituents in the set, the selection criteria used by a receiving system to choose an aggregate product provides evidence that each of these exchanges in the set represent a different *niche*, and the emergence of the set represents one possible process through which *cultural niche construction* may occur as outlined in the fifth extended criteria of CCE as defined by Mesoudi and Thornton (2018).

Couto and Sandaker (2016) revisited Skinner’s conceptualization of the process of selection involved in the evolution of cultures and noted that the object of selection was not clearly defined. They recounted an exchange between evolutionary theorist Richard Dawkins (1984) and Skinner (1988) where this issue was a topic of focus. Dawkins questioned if the entities selected via Skinner’s third level of selection were cultural practices or were the cultures as a whole (of which the cultural practices were part). Skinner’s response was that within-groups practices are selected and transmitted (the production process, or mechanism), whereas between-cultures features such as social systems and technological methods are the objects of selection. By way of analogy, Skinner (1988) likened the evolution of cultural practices to the evolution of particular organs (like the heart, stomach, leg, etc.), whereas the evolution of cultures was likened to the survival and evolution of the whole organism of the species because of the functional and survival advantages that its particular organs might provide in a given environment.

With this exchange as context, Couto and Sandaker (2016) considered the metacontingency and Glenn’s (2004) conceptualization of cultural selection, concluding that the metacontingency (selecting the IBCs that produced the aggregate product) was a within-groups process and, as a result, cultural selection is a particular process that accounts for the selection of cultural practices and thus does not rise to the between-groups, third level of selection through which the evolution of cultures (or what they called the *selection of cultures*) is achieved. Couto and Sandaker asserted that the selection of cultures involves the selection of environmental settings, or cultural-social environments.

Krispin (2017) proposed that autofacilitative sets like the culturant hypercycle meet the criteria that Couto and Sandaker (2016) defined for a process of selection by consequence through which the selection of cultures might be achieved. Although each culturant in the hypercycle recurs because of the reinforcing consequences that it receives upon its selection by the subsequent culturant, the overall set is accelerated due to the positive feedback loop established by centripetality of the set overall. The contingencies of local reinforcement change the rates of recurrence for each member, whereas the feedback dynamics of the set change the contingencies to enrich the rates of reinforcement for each member of the set.

**Fig. 2:**
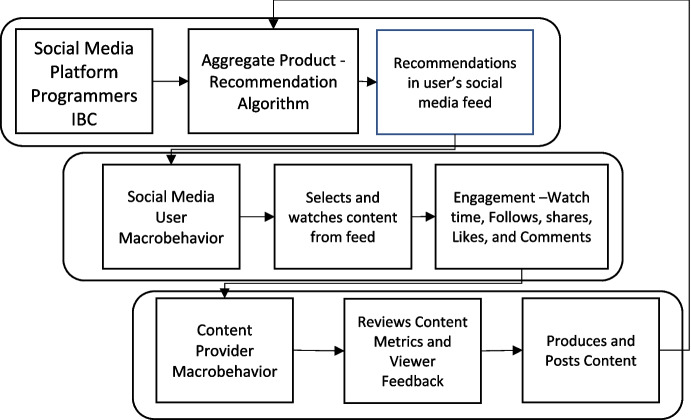
Social Media Culturo-Behavioral Hypercycle. *Note*. Outputs generated by the recommendation algorithm populate the social media feed of a social media user (culturant level). The social media user selects content with which they engage from the feed (operant level). Content providers monitor engagement statistics from the social media users who engage with their content, and produce additional similar content (either operant or culturant level), which feeds back into the recommendation algorithm. When outputs from each stage fit with the selection criteria of each subsequent stage, a positive feedback loop is established and centripetality is exhibited among the set, producing “viral” phenomena. (Alavosius et al., 2024)

Krispin (2019) also asserted that such positive feedback loops might be established via the intermingling of operant and culturant levels of behavior—the culturo-behavioral hypercycle. Alavosius et al. (2024) provided an example of such a set, explaining how “viral” phenomena in social media may emerge from cross-level interactions among culturants and operants (see Figure 2). The centripetality characteristic such as may be exhibited in some interactions on social media is exhibited on several levels. First, individual social media posts can go viral and explode in terms of the number of viewings via such feedback loops. Second, the most viewed social media posts generate social media trends, with similar posts produced by a multitude of content providers. Third, the positive-feedback loops generated by these viral phenomena attract many new social media users and content providers to the social media platform, and we can see explosive growth on the platform level, as has been observed through the growth of Facebook and, more recently, social media platforms like Instagram and TikTok.

Next, we will turn our attention to reconsidering the revised concept of the cultural cusp as presented by Glenn and Malott (2024) in their conference presentation in light of the current discussion. They proposed the following definition for the cultural cusp: Dynamic ecosystems resulting in a novel product that leads to significant cultural change in social, political, and economic environments. Given the concepts explored in this article, we might understand this as being a property of complex behavioral systems, particularly the nonlinear relationships between changes at the boundary conditions of such systems and the resulting changes within the system. There are several ways in which this nonlinear relationship might manifest.

One causal explanation for why (and how) complex behavioral systems might exhibit the behavior associated with a cultural cusp (significant cultural change resulting from a novel product) is directly related to their self-organizing tendencies as they expand into their adjacent possibles. For example, the transition from metaculturants to closed-loop, culturant hypercycles might present such a case. When the “final link” is made to close a metaculturant chain, and a culturant (or culturo-behavioral) hypercycle is formed, the collective acceleration of all of activity within the set of culturants may be large enough to cause the kind of significant cultural change to which Glenn and Malott refer.

The larger effects of the formation of a new culturant- or, in a more general sense, culturo-behavioral hypercycle may also include the draining of resources that may have previously sustained other cultural practices (whether in a culturo-behavioral hypercycle, or in a series of relatively-independent cultural practices), reducing the frequency with which those other practices occur and thereby compounding the impact of the formation of the new culturo-behavioral hypercycle on the overall cultural dynamics.

A second manner in which significant cultural impacts may also occur via cultural cusps may occur when a culturo-behavioral hypercycle is interrupted or disrupted in some way. For example, in some cases, the interruption or slowing of even individual links in a culturo-behavioral hypercycle can result in the collapse of the centripetal dynamics of the set, freeing up resources that might fuel rapid growth in other culturo-behavioral hypercycles.

To the extent that the constituent members of cultural ecosystems are interlocked with each other, the ripple effects of these kinds of changes can be quite extensive, and it is essentially impossible to predict exactly how such cascading cultural changes may unfold. The power-law patterns of change exhibited by such complex systems tell us that much larger changes (both growth and collapse) than we might intuitively expect will happen significantly more often than we might expect (see, for example, the discussion of *extremistan* and *mediocristan* in *The Black Swan* by Taleb, 2007/2010). In all complex systems in which positive feedback dynamics are possible, small local changes can produce much larger changes that sweep across the system, leading to nonlinear changes in dynamic responses to local perturbations at the boundaries of the set and fluctuations within the set. Given the interconnectivity now present in modern-day society via the internet and social-media platforms (themselves examples of aggregate products involved in culturo-behavioral hypercycles), cultural volatility is increasing.

Finally, we can return to the threefold factors that Wasserman (2021, 2024) asserts may be sufficient to account for the diversity of behavioral practices and products that we observe—context, consequence, and coincidence. We have seen how the definition of context expands as behavioral systems explore their adjacent possible and the types of behavioral interactions become more complex. The niches that are formed as operant behavior of individuals initially reinforced by directly occurring consequences transitions into socially reinforced operants, then culturants and metaculturants, and ultimately to culturo-behavioral hypercycles of increasing diversity and complexity determine fitness using selection criteria that can become quite distantly related to the fundamental tenet of survival of the individual organism originally defined by Darwin in natural selection. It becomes increasingly more difficult to define what is meant by “survival”—are we speaking of survival of the individual, survival of the behavior/practice, survival of the organization, or survival of the culture?

Consequences, or more generally, processes of selection by consequences extend beyond the original processes of natural and operant selection, now including processes that select entire sets of aggregate products and the processes that produce them in the form of culturant hypercycles, and sets of interactions between individuals and these aggregate products in the form of culturo-behavioral hypercycles. The web of interconnected behavioral ecosystems is quite complex indeed. In the examples that we have considered across varying levels of combinatorial systems, we have seen that the mechanics of such systems can be vastly different, whereas the dynamics of such systems can be similar. The dynamic principles that underlie self-organization apparently hold across systems of vastly different structures and levels.

Last, although it is clear that variation at the behavioral level due to operant selection can and does play a role in CCE, it should also be clear that there are other processes of selection by consequence like those that result in culturant and culturo-behavioral hypercycles, followed by the top-down selective pressures on their constituents that those systems exert that has led to the development of the present levels of diversity and complexity observed in culturo-behavioral systems today. Once a cultural niche is formed, the dimensions of fitness that lead to selection can be explored and defined, and, once those criteria are defined, there is a target against which variation can be compared—the concept of improvement begins to have meaning. As Wasserman (2021, 2024) presented, these improvements may have been originally due to true random variation and coincidence but have transitioned to “tinkering” and ultimately to engineering solutions as cultures have expanded into their respective adjacent possibles and produced cumulative artifacts on which future improvements are built. There is still room for true random variation—perhaps at the forefront of the expansion into the adjacent possible, where the fitness criteria of as-yet-defined niches are unfolding—but the cumulative record of learning kept in our various languages has fundamentally changed the game and accelerated the rate at which we can build upon a foundation of learning that is itself rapidly expanding.

## Data Availability

No datasets were generated or analysed during the current study.

## References

[CR1] Alavosius, M. P., Rakos, R. F., & Krispin, J. V. (2024). Three recent books on social media manipulation, misunderstanding science, and caring about future people: Opportunities for behavior analysts. *Behavior & Social Issues,**33*, 909–924. 10.1007/s42822-024-00185-2

[CR2] Bianconi, G., Arenas, A., Biamonte, J., et al. (2023). Complex systems in the spotlight: Next steps after the 2021 Nobel Prize in Physics. *Journal of Physics: Complexity,**4*(1), 1–28. 10.1088/2632-072X/ac7f75

[CR3] Biebracher, C. K., Nicolis, G., & Schuster, P. (1995). *Self-organization in the physico-chemical and life sciences.* Report No. EUR 16546 prepared for the Directorate General XII for Science, Research, and Development, European Commission. Brussels, Belgium.

[CR4] Catania, C. . (1973). The concept of the operant in the analysis of behavior. *Behaviorism,**1*(3), 103–116.

[CR5] Cep, C. (2019). The real nature of Thomas Edison’s genius. *The New Yorker.*

[CR6] Couto, K. C., & Sandaker, I. (2016). Natural, behavioral and cultural selection-analysis: An integrative approach. *Behavior & Social Issues,**25*, 54–60. 10.5210/bsi.v25i0.6891

[CR7] Daniels, A. C. (1989). *Performance management.* Performance Management.

[CR8] Darwin, C. R. (1958). *The autobiography of Charles Darwin 1809*–*1882.* Collins. (Original work published 1887)

[CR9] Dawkins, R. (1984). Replicators, consequences, and displacement activities. *Behavioral and Brain Sciences,**7*(04), 486–487. 10.1017/S0140525X00026790

[CR10] Glenn, S. S. (2004). Individual behavior, culture, and social change. *The Behavior Analyst,**27*, 133–151. 10.1007/BF03393175 22478424 10.1007/BF03393175 PMC2755396

[CR11] Glenn, S. S., Malott, M. E., Andery, M. A. P. A., Benvenuti, M., Houmanfar, R. A., Sandaker, I., Todorov, J. C., Tourinho, E. Z., & Vasconcelos, L. A. (2016). Toward consistent terminology in a behaviorist approach to cultural analysis. *Behavior and Social Issues,**25*, 11–27. 10.5210/bsi.v25i0.6634

[CR12] Glenn, S., & Malott, M. (2024). Behavior and cumulative cultural evolution. 2024 Theory and Philosophy Conference, Association of Behavior Analysis, International, Chicago, IL.

[CR13] Herrnstein, R. J., & Vaughn, W. (1980). Melioration and behavioral allocation. In J. E. R. Staddon (Ed.), *Limits to action: The allocation of individual behavior* (pp. 143–176). Academic Press.

[CR14] Hudson, G. . (2000). From Social Darwinism to self-organization: Implications for social change theory. *Social Service Review,**74*, 533–559. 10.1086/516424

[CR15] Hunter, C. S. (2012). Analysing behavioral and cultural selection contingencies. *Revista Latinoamericana de Psicología,**44*(1), 43–54.

[CR16] Kauffman, S. A. (2000). *Investigations*. Oxford University Press.

[CR17] Krispin, J. (2017). Positive feedback loops of metacontingencies: A new conceptualization of cultural-level selection. *Behavior & Social Issues,**26*, 95–110. 10.5210/bsi.v26i0.7397

[CR18] Krispin, J. V. (2019). Culturo-behavioral hypercycles and the metacontingency: Incorporating self-organizing dynamics into an expanded model of cultural change. *Perspectives on Behavior Science,**42*(4), 869–887. 10.1007/s40614-019000212-3 31976464 10.1007/s40614-019-00212-3 PMC6901668

[CR19] Marr, J. (1996). A mingled yarn. *The Behavior Analyst,**19*, 19–33. 22478237 10.1007/BF03392736 PMC2733601

[CR20] Marshall, S. M., Mathis, C., Carrick, E., Keenan, G., Cooper, G. J. T., Graham, H., Craven, M., Gromski, P. S., Moore, D. G., Walker, S. I., & Cronin, L. (2021). Identifying molecules as biosignatures with assembly theory and mass spectrometry. *Nature Communications,**12*, Article 3033. 10.1038/s41467-021-23258-x 34031398 10.1038/s41467-021-23258-x PMC8144626

[CR21] Mattaini, M. A. (2006). Will cultural analysis become a science? *Behavior & Social Issues,**15*, 68–80. 10.5210/bsi.v15i1.380

[CR22] Mattaini, M. A. (2007). Editorial: Technical language in cultural analysis. *Behavior & Social Issues,**16*, 1–4. 10.5210/bsi.v16i1.1811

[CR23] Mesoudi, A., & Thornton, A. (2018). What is cumulative cultural evolution? *Proceedings of the Royal Society B: Biological Sciences,**285*, Article 20180712. 10.1098/rspb.2018.0712 10.1098/rspb.2018.0712 PMC6015846 29899071

[CR24] Nia, H. T., Jain, A. D., Liu, Y., Alam, M.-R., Barnas, R., & Makris, N. C. (2015). The evolution of air resonance power efficiency in the violin and its ancestors. *Proceedings of the Royal Society A: Mathematical, Physical and Engineering Sciences,**471*, Article 20140905. 10.1098/rspa.2014.0905 10.1098/rspa.2014.0905 PMC4353046 25792964

[CR25] Prigogine, I. (1980). *From being to becoming: Time and complexity in the physical sciences*. Freeman.

[CR26] Prigogine, I. (1996). *The end of certainty: Time, chaos, and the new laws of nature*. Free Press.

[CR27] Prigogine, I., & Stengers, I. (1984). *Order out of chaos: Man’s new dialogue with nature.* Bantam Books.

[CR28] Sandaker, I. (2009). A selectionist perspective on systemic and behavioral change in organizations. *Journal of Organizational Behavior Management,**29*(3–4), 276–293. 10.1080/01608060903092128

[CR29] Skinner, B. F. (1974). *About behaviorism*. Vintage Books.

[CR30] Skinner, B. F. (1981). Selection by consequences. *Science,**213*, 501–504. 10.1126/science.7244649 7244649 10.1126/science.7244649

[CR31] Skinner, B. F. (1988). Responses to commentaries. In A. C. Catania & S. Harnad (Eds.), *The selection of behavior: The operant behaviorism of B. F. Skinner* (p. 37). Cambridge University Press.

[CR32] Sterman, J. (2000). *Business dynamics: Systems thinking and modeling for a complex world.* Irwin McGraw-Hill.

[CR33] Taleb, N. N. (2007/2010). *The black swan: The impact of the highly improbable*. Random House.

[CR34] Ulanowicz, R. E. (2009). *A third window*. Templeton Foundation Press.

[CR35] Walker, S. I. (2024). *Life as no one knows it: The physics of life’s emergence.* Riverhead Books.

[CR36] Wasserman, E. A. (2021). *As if by design: How creative behaviors really evolve.* Cambridge University Press.

[CR37] Wasserman, E. (2024). Unhinging design from Darwinian and Skinnerian selection. 2024 Theory and Philosophy Conference, Association of Behavior Analysis, International, Chicago, IL.

[CR38] Zeiler, M. D. (1992). On immediate function. *Journal of the Experimental Analysis of Behavior,**57*(3), 417–427. 10.1901/jeab.1992.57-417 16812660 10.1901/jeab.1992.57-417 PMC1323239

